# The evolution of the posterior approach in hip surgery: Back to Langenbeck

**DOI:** 10.1007/s00423-026-03993-9

**Published:** 2026-02-17

**Authors:** Diederik R. de Boer, Roelina Munnik – Hagewoud, Frank F. A. IJpma, Pieter B. A. A. van Driel, Harmen B. Ettema

**Affiliations:** 1 Department of Orthopedics, University of Groningen, University Medical Center Groningen, Groningen, the Netherlands; 2 Department of Orthopedics, Isala Hospital, Zwolle, the Netherlands; 3 Department of Trauma Surgery, University of Groningen, University Medical Center Groningen, Groningen, the Netherlands

**Keywords:** Hip surgery, Posterior approach, History, Bernhard von Langenbeck, Minimal invasive surgery

## Abstract

**Purpose:**

The posterior approach is a proven approach in hip surgery. Over the past 150 years, we have seen many variations of posterior approaches arising from predecessors, with Dr. Bernhard von Langenbeck (1810–1887) as the founder of the modern posterior approach. The aim of this historical review is to understand the evolution of the posterior approach from its ‘birth’ with von Langenbeck to modern approaches in hip surgery.

**Methods:**

We analysed von Langenbeck’s original manuscript, as well as literature of subsequent approaches.

**Results:**

Over the past two centuries, surgical approaches to the hip joint have adapted to the changing needs and goals of surgeons and patients. Von Langenbeck developed a minimal invasive posterior approach in 1868 for femoral head resection of injured soldiers. According to subsequent surgeons, the perioperative view on the hip joint and surrounding structures was too minimal to reduce the number of complications, implement the first prostheses and fix fractures. As a result, surgical approaches became more invasive. Around 1900, Kocher and Moore further developed von Langenbeck’s approach more distally into the iliotibial band. After World War II, the Judet brothers extended their incision proximally to the pelvis to fix acetabular fractures. With the changing western society in the early 2000s, the demand for minimally invasive approaches in total hip arthroplasty grew. Superior approaches were developed and show many similarities to von Langenbeck’s approach 150 years earlier.

**Conclusion:**

With the advent of minimally invasive superior approaches, the evolution of posterior approaches in hip surgery is *Back to Langenbeck*!

## Introduction

Orthopaedic literature describes a wide variety of surgical approaches to reach the hip joint [1]. Many of these approaches are used in daily practice and somehow derived from just a few ‘*original approaches*’. One of those is the posterior approach, which is often considered the gold standard in total hip replacement surgery. Its origins can be traced back to Dr. Bernhard von Langenbeck, a German surgeon who introduced this approach in 1868 [2]. As one of his many medical discoveries, his efforts on the posterior approach were of great value for the field of orthopaedic and trauma surgery. Von Langenbeck’s posterior approach to the hip is seen as the starting point for the variety of modified posterior approaches such as those by legendary surgeons like Kocher and Moore. To fully understand the logic behind the different modified posterior approaches, it is important to go back in history and analyse the evolution of the posterior approach in hip surgery. By studying history, we gain insights into the patterns that shape the future.

This historical review aims to examine the origin and evolution of the posterior approach, and its incorporation into modern surgical approaches to the hip joint. For this purpose, we analysed von Langenbeck’s original manuscript titled ‘*Schussfracturen der gelenke und ihre behandlung’* from the *Bayerische Staatsbibliothek* (1868) [2], as well as literature of modified approaches by key surgeons. The results are presented in three parts in this historical narrative review, followed by a general discussion. The first part describes the life of von Langenbeck and the birth of the posterior approach. The second part consists of von Langenbeck’s main successors and their modifications to the posterior approach. Part three discusses modern trends in posterior approaches. This review is not intended to summarize all modified approaches but will focus on understanding the evolution of the posterior approach over the past 150 years.

## Results

### Part 1. The birth of the posterior approach to the hip joint

Bernhard Rudolph Conrad von Langenbeck (1810–1887, Fig. 1a) was born in Padingbüttel, a small town in Germany near the North Sea [3]. His uncle, Conrad J. M. Langenbeck, was a prominent ophthalmologist and professor of surgery at the University of Göttingen [4, 5]. With the guidance of his uncle, Bernhard started studying medicine at the same university in 1830. Soon after graduating with honours in 1834 with his thesis on the structure of the retina, a postdoctoral scholarship allowed him to study in London and Paris under world-renowned surgeons such as Sir Benjamin Brodie and Sir Astley Cooper [3]. Eventually, von Langenbeck returned to Germany in 1840 and became professor of physiology and pathological anatomy in Göttingen [4]. In 1842, he left Göttingen “*to investigate the mysteries of the human body*” as a professor of surgery at both Universities of Kiel and Berlin [4].

**Fig. 1:**
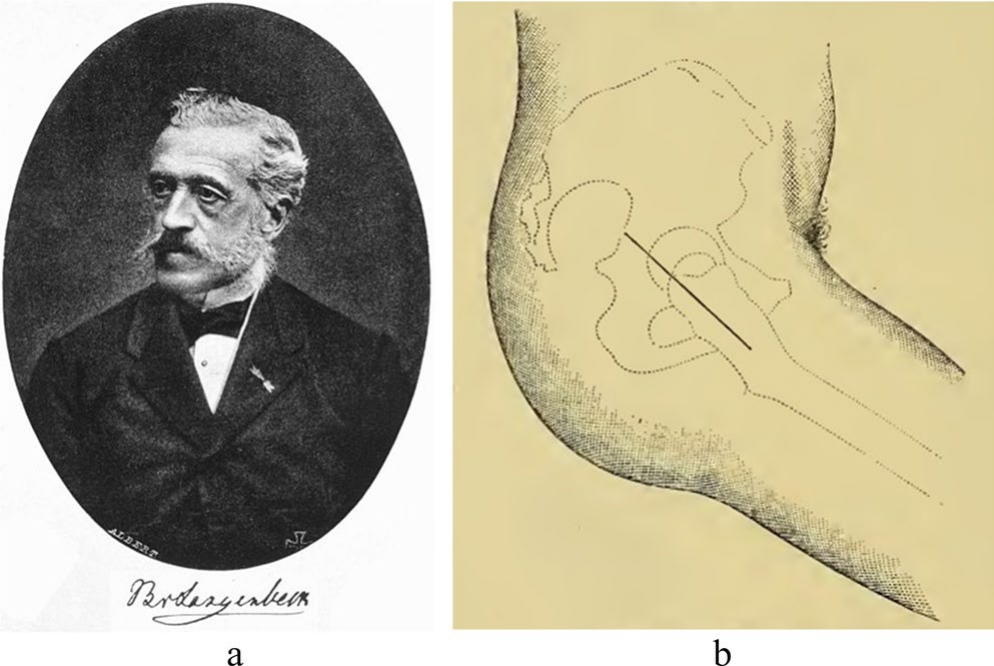
Bernhard von Langenbeck’s (**a**, public image) longitudinal incision to reach the hip joint (**b**, reprinted from Esmarch, 1884) [9]

At both universities, von Langenbeck was an immensely popular lecturer. He possessed *“a refined face and a noble look in his eye*,* he was an aristocrat by nature and in feeling”*, according to Walter Platt, one of his students [3]. Many of his pupils became excellent and well-known surgeons, such as Trendelenburg, Billroth and Hueter. Billroth even regarded him as a father, *“His name is written in golden letter not only in the Book of History but in the Book of Love of all his pupils and friends”* [3]. Good collaboration between von Langenbeck and his pupils resulted in the foundation of the *Archiv für Klinische Chirurgie* (currently known as *Langenbeck’s Archives of Surgery*) in 1860 [4]. This scientific journal is now known as the oldest surgical journal worldwide [6].

During the Holstein Wars (1848 & 1863), Austro-Prussian War (1866) and Franco-Prussian War (1870), von Langenbeck served as a trauma surgeon on the battlefield. Here, von Langenbeck sought ways to reduce amputations and improve the outcomes for soldiers with hip joint injuries. He was able to operate on many war casualties, optimizing his technique of subperiosteal joint resection [7]. By preserving the periosteum, the insertion of tendons to the diaphysis could be maintained. Following the resection of the epiphysis, von Langenbeck believed that a functional joint would ultimately persist, allowing front-line officers to resume active duty after their injury.

In an attempt to reduce the number of infected war wounds caused by gunshot fire around the hip joint, von Langenbeck used a technique described by himself as the ‘*longitudinal incision*’ for resecting the femoral head (which he called ‘hip joint resection’, Fig. 1b) [2]. He was not content with the curved incision of Anthony White (1782–1849), which von Langenbeck had used for years [2]. White performed the first hip excision in 1821, although he did not report his approach in literature due to his successful practice [2, 8]. White’s curved incision begun at the posterior superior iliac spine, carried around the greater trochanter and descended distally with the leg in extension [9]. According to von Langenbeck, this approach was too harmful for the patient [2].

**Fig. 2:**
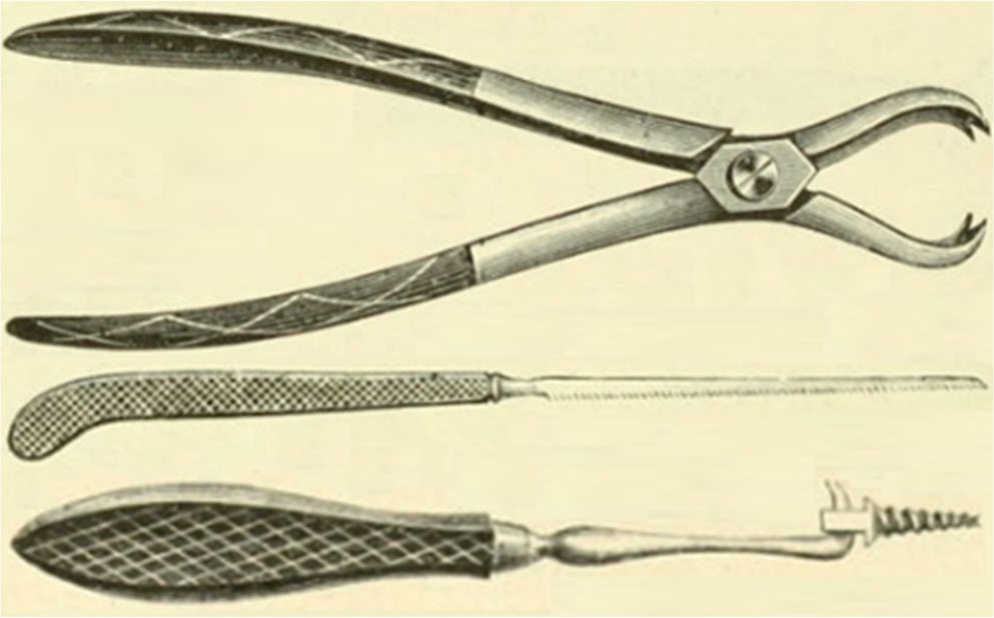
Von Langenbeck’s forceps, jab saw and Tirefond (reprinted from Esmarch, 1884) [9]

Therefore, von Langenbeck started practicing another approach as from 1866, performing the procedure twice on ‘live subjects’ and perfecting it on cadaver specimens [2]. With 222 words, von Langenbeck first described the fundamentals of his approach in his 1868 manuscript. During von Langenbeck’s approach, the hip was flexed 45 degrees and a straight incision of approximately 5 inches (12 cm) was made from the middle of the greater trochanter towards the posterior superior iliac spine in line with the axis of the femur [2]. The gluteal muscles were carefully split preserving the distal connection with the thigh fascia and periosteum. According to the manuscript (1877) of his colleague at the university of Kiel, Dr. Friedrich von Esmarch (1823–1908), von Langenbeck detached the piriformis and the conjoined tendon, along with the gluteus minimus and gluteus medius, carefully preserving their periosteal attachment [9]. The joint capsule was incised longitudinally and the obturator externus muscle was released, while maintaining the connection with the periosteum. To cut the ligamentum teres, von Langenbeck inserted a small straight knife between the femoral head and the acetabulum, allowing the release of the femoral head. He removed the femoral head using a hook forceps, thin jab saw and a ball-screw or ‘Heine’s Tirefond’ (Fig. 2) [7]. After removal of the femoral head, the detached muscles were not repaired, and the wound was closed.

**Fig. 3:**
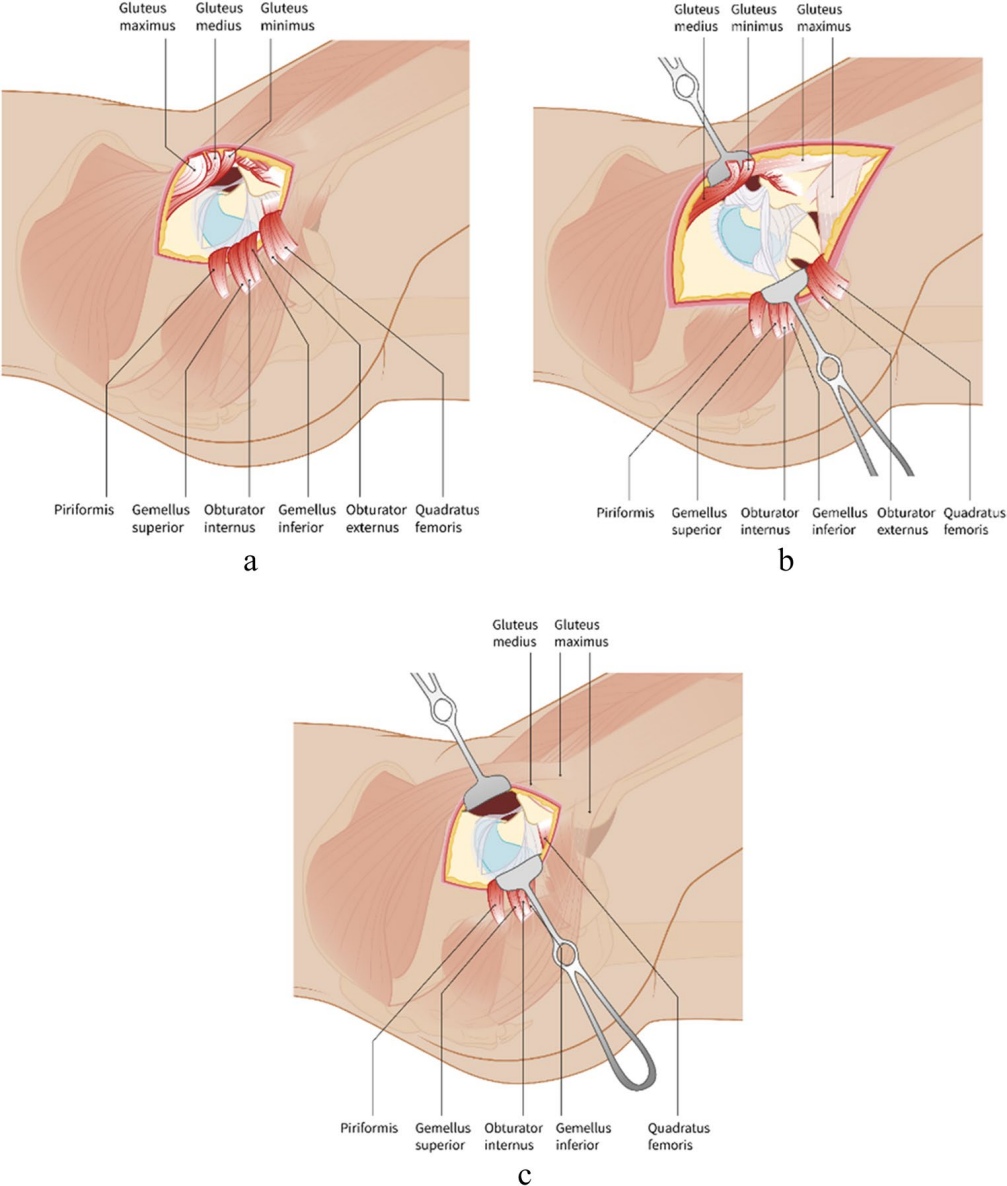
**a**) The longitudinal approach of von Langenbeck from superior trochanter towards posterior superior iliac spine is superior to the iliotibial band. All external rotators, as well as the gluteal muscles, are released. Von Langenbeck uses a superior capsule incision. **b**) The incision of the oblique approach of Kocher is extended distally into the iliotibial band. Modern versions of the Kocher-Langenbeck approach save the gluteal muscles, which were released in the original description of the approach. In different versions, different external rotators were released. **c**) The incision of the direct superior approach is similar to Langenbeck’s approach, superior to the iliotibial band. In the direct superior approach, the piriformis muscle and conjoined tendon are the only released muscles

Eight years (including the Franco-Prussian war) later, having performed the approach numerous times, von Langenbeck further reflected on the procedure in his book ‘*Chirurgische Beobachtungen aus dem Kriege*’ [7]. Von Langenbeck did report some problems he encountered using his approach. He believed that hip resection should not be performed on the battlefield, as patient transport after surgery was challenging to avoid hip flexion contractures. Since no major vessels were encountered, von Langenbeck observed only minimal bleeding. Nevertheless, he described one patient with excessive arterial bleeding from perforating and gluteal arteries, which he thought was associated with venous thromboembolism. Further, he described having difficulty in a case with many bone fragments penetrating the muscles [7]. Overall, he considered the exposure of the joint excellent and with the major advantage of the preservation of muscles in their attachment to the periosteum. Von Langenbeck’s ‘*longitudinal approach*’ is considered the original description of posterior approach to the hip joint, as we know it today (Fig. 3a).

### Part 2. Down into the iliotibial band and up the pelvis

A new era dawned when surgeons called for more visibility to the hip with less damage to muscle, nerve and bone than provided by von Langenbeck’s approach [10]. Two options were available: (1) proximal extension of the approach superiorly, either anteriorly or posteriorly; or (2) distal extension into the iliotibial band to expose the femur. The first to embark on the latter was Dr. Theodor Kocher (1841–1917, Fig. 4a). Kocher was born and raised in Bern, Switzerland and graduated *summa cum laude* from the University of Bern Medical School in 1865 [11]. Kocher visited several clinics in Europe after his graduation including von Langenbeck’s clinic in Berlin. Ironically, however, Kocher was denied admission for postgraduate surgical training because of his Swiss nationality [11]. When he returned to Bern, Kocher became, with support of von Langenbeck [12], professor of surgery and chief of surgery at the University Medical Centre of Bern at the age of thirty until he died in 1917 at the age of seventy-six. As a surgeon, Kocher was precise and disciplined, advocating a systematic approach to surgical procedures. He implemented standardized wound dressing and standard instruments in surgery. Along with a well thought-out, stepwise approach to the surgery, this allowed Kocher to reduce mortality associated with thyroid surgery from 12.8% in 1882 to 0.5% in 1917 [13]. This exceptional achievement was rewarded with the Nobel Prize in 1909. His book published in 1892, simply called ‘*Chirurgische Operationslehre’*, became a worldwide standard work [14].

**Fig. 4:**
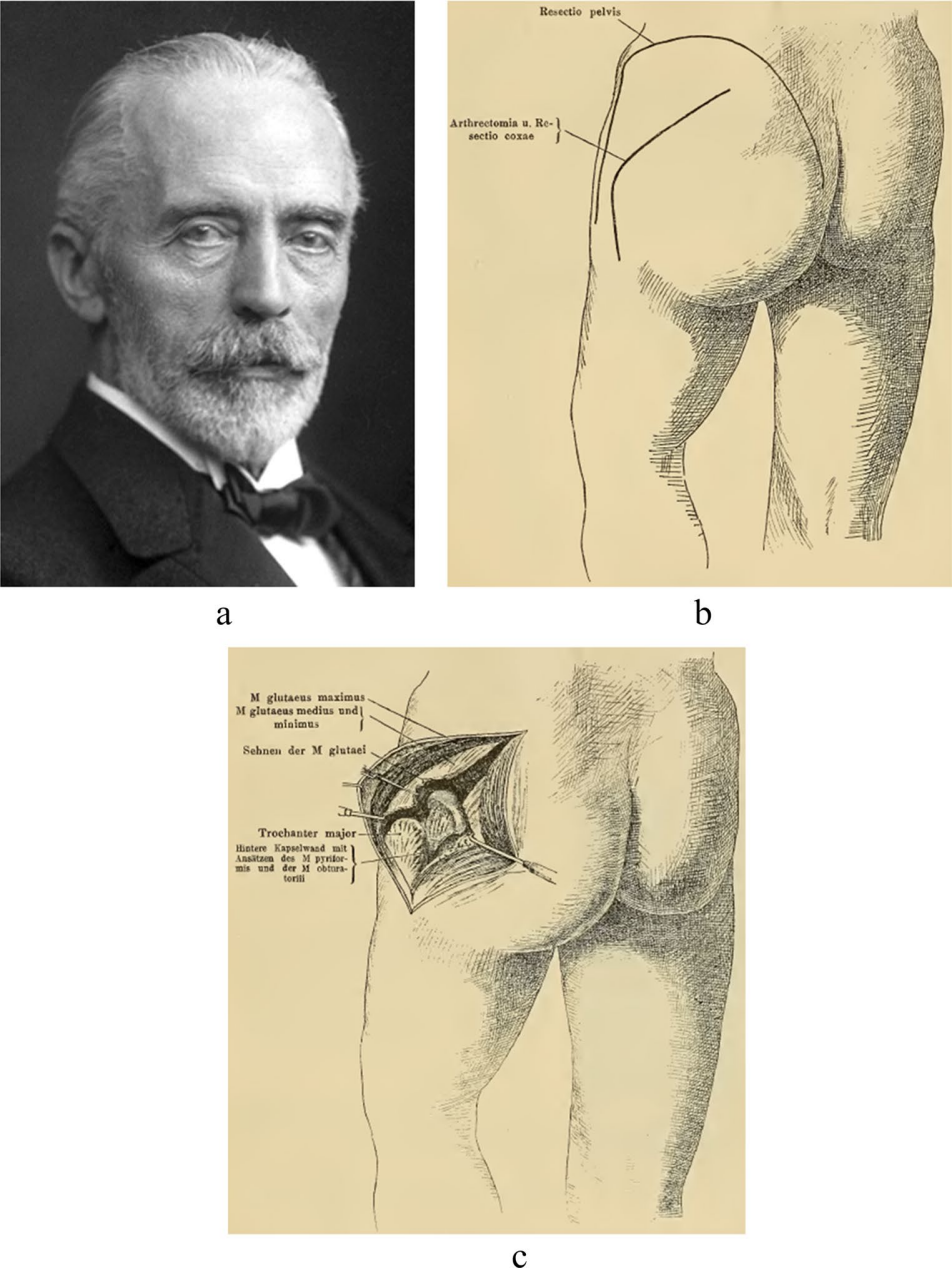
Theodor Kocher’s (**a**, public image) oblique approach (**b**, **c**, reprinted from Kocher, 1892) [10]

In this book, Kocher also described a modification to von Langenbeck’s longitudinal approach, which he particularly used for removal of tubercular tissue [10]. Kocher believed that the exposure to the hip joint in von Langenbeck’s approach was too limited with too much injury to the muscles, nerves and bone [10]. Kocher’s approach involved angulating the incision to a curved one, which was extended distally (Fig. 4b/4c). The incision started a few centimetres distally and laterally to the posterior superior iliac spine. Kocher progressed the incision over the greater trochanter towards lateral aspect of the greater trochanter’s base. The iliotibial band, which had been preserved by von Langenbeck, was divided, along with the gluteus maximus. Subsequently, the capsule was incised above the piriformis. The leg was flexed and rotated outwards, followed by separation of the gluteus medius and minimus from the greater trochanter. The piriformis muscle as well as the other short external rotator muscles were detached from the greater trochanter. The hip capsule was incised and the ligamentum teres was divided to provide dislocation of the head. Kocher said: “*It is a further development of Langenbeck’s method by the oblique incision*” [10]. Kocher was therefore the first surgeon to describe extending the incision into to iliotibial band to further expose the hip (Fig. 3b).

Dr. Alexander Gibson (1883–1956) was the first advocate of the posterior approach across the ocean in North America. Gibson’s 1950 article shows his faith of the ‘*outstanding advantages*’ that this approach provides [15]. Gibson was born, raised and educated in Great Britain. In 1913, he accepted a position as Chair of Anatomy at the University of Manitoba, Winnipeg, Canada and was subsequently appointed professor at the same university [16]. He introduced his version of Kocher’s posterior approach to North America, where it had been relatively unknown until then. Gibson shifted the skin incision more laterally and extended it further distally than Kocher. Furthermore, the gluteal fascia was divided after skin incision, allowing retracting and preservation of the gluteus maximus. The other gluteal muscles and the short external rotators could be detached and the capsule incised to dislocate and replace the head of the femur. Gibson hypothesized that sparing of the gluteus maximus muscle would reduce instability of the hip. The importance of the other gluteal and short external rotators muscles only became apparent later, despite Kocher already recognizing the important abductor function of the gluteal muscles [10].

**Fig. 5:**
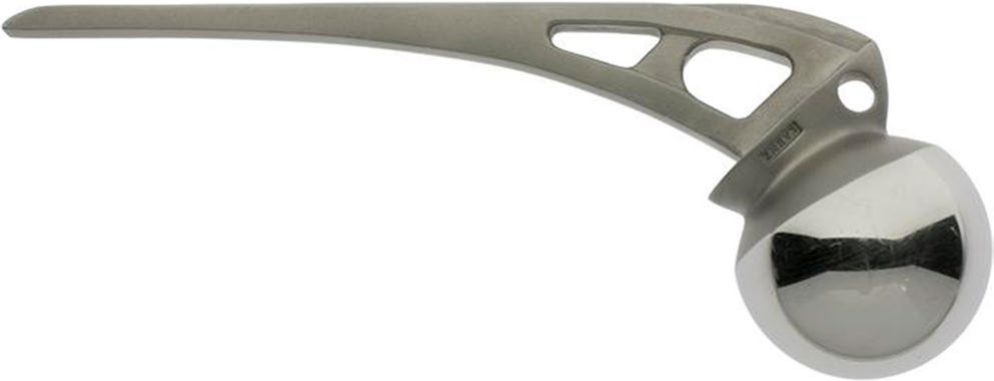
Austin Moore’s prosthesis with a large femoral head made of Vitallium as used in 1957 (Image from Science Museum Group Collection) [38]

The modern posterior approach to reach the hip joint bares most resemblance to the approach introduced by Dr. Austin Moore (1899–1963) [17]. Moore was an American orthopaedic surgeon who performed the first metallic hip replacement at Columbia Hospital, South Carolina with his own designed femoral prosthesis and large femoral head made of Vitallium (Fig. 5) [18]. He reported his approach in 1957 as the “Southern approach”, named for two reasons: (1) the skin incision was shifted more posteriorly—metaphorically described as “southern”; and (2) he was based in the southern United States [17, 19]. The incision of the Southern approach starts approximately two inches distally and posteriorly of the posterior inferior iliac spine with the leg in 45 degrees flexion. The incision courses along the posterior border of the greater trochanter for about four inches distally. The fascia lata and iliotibial band are incised along with the gluteus maximus. The short external rotator muscles are detached from the greater trochanter. If necessary, the quadratus femoris could be partially incised. A distal capsulotomy is performed and the hip will be dislocated. The most innovating aspect about Moore’s approach was the preservation of not only the gluteus maximus but also the gluteus medius and minimus - contrary to his predecessors.

Due to the rising incidence of acetabulum fractures associated with the increasing number of automobiles in Paris, Robert Judet (1909–1980), Jean Judet (1905–1995) and Émile Letournel (1927–1994) sought to develop a classification system and surgical approach for management of these fractures [20]. While the Southern approach allowed adequate exposure of the hip joint, it did not provide an extended surgical field for the treatment of acetabular fractures. The Judet brothers and Letournel utilized the approaches of Kocher and von Langenbeck and developed their own “*Kocher-Langenbeck approach*” in 1955 [20, 21]. The upper part of their incision was extended towards the posterosuperior iliac spine until adequate visualization of the acetabulum was achieved. After dividing the iliotibial band and splitting the gluteus maximus proximally, the femoral shaft insertion of the gluteus maximus distally was incised to reduce muscular tension, allow for easier retraction of the gluteus maximus, and subsequently improve exposure of the posterior column. The piriformis and conjoint tendon insertions were incised. Other short external rotators were resected depending on exposure needs. As of the writing of this manuscript, the Judet-Letournel classification of acetabular fractures and their surgical approaches (Kocher-Langenbeck and ilioinguinal) are still considered foundational techniques for the reduction and fixation of acetabular fractures [1, 22, 23].

### Part 3. Superior approaches: saving the iliotibial band again

At the end of the 20th century, the focus of healthcare rapidly shifted due to increasing demands in western society. Healthcare costs were rising, average age and incidence of obesity were increasing, surgeries were performed on relatively younger and healthier patients, and the number total hip arthroplasties, along with the patient expectations, was increasing [24, 25]. Faster discharge and rehabilitation were desired [25, 26]. In the late 1990s, the concepts of mini-incision and minimally invasive surgery emerged, initially defined by smaller skin incisions, typically not exceeding 10 centimetres, with the goals of reducing hospital stay and accelerating postoperative recovery [27, 28]. However, it soon became evident that the length of the skin incision was less critical than the preservation of deep anatomical structures essential for hip stability and function, including the short external rotators, obturator externus, and hip abductors. Consequently, surgical innovation shifted towards approaches that minimize trauma to these musculo-tendinous structures to maintain native hip biomechanics [27, 28]. By shifting the surgical approach superiorly, the iliotibial band could be spared, providing secondary lateral hip stability and potentially preventing a Trendelenburg gait [29]. Similarly, alternative iliotibial band–sparing approaches, such as the anterior approach, have also reported a lower incidence of Trendelenburg gait [30, 31], demonstrating the benefits of preserving key anatomical structures beyond simply reducing skin incision length.

Dr. Stephen Murphy, an orthopaedic surgeon at New England Baptist Hospital in Boston, United States, was one of the first to develop a minimally invasive superior approach, the SuperCap, in 2004 [32]. The base incision of the SuperCap is limited to 6–8 cm superior to the greater trochanter in line with the femoral shaft. The gluteus maximus is split to reveal the gluteus medius and the piriformis (released if necessary). These were retracted anteriorly and posteriorly, respectively. After retracting the gluteus minimus anteriorly, a superior capsulotomy is performed, allowing the development of an anterior flap. The superior part of the head and neck were removed to allow femoral broaching. The remaining neck and head remain in situ to maintain stability, until femoral component is placed after which they are subsequently resected. The approach necessitated the development of 45° angled reamers and Z-shaped impactors to prepare the acetabulum and allow impaction of the acetabular cup while existing the initial incision. After insertion of the acetabular component, the capsule and released muscles were repaired.

To avoid the need for angled reamers, Dr. Brad Penenberg, an orthopaedic surgeon in Beverly Hills, United States, introduced a new approach in 2008 using a cannula, which he named “percutaneously assisted total hip arthroplasty” (PATH) [33]. Compared to SuperCap, the incision was made more posteriorly. The piriformis is always released in PATH and the capsulotomy is made as inferior as possible. The femoral head is dislocated before the femoral neck is cut and the femur is ready for broaching. Acetabular reaming is performed with assistance of a cannula to minimise the incision and limit soft-tissue damage. The cannula is placed just behind the posterior edge of the femoral shaft. A reamer basket is placed in the acetabulum connected with the reamer driver though the cannula.

In an effort to optimize the SuperCap and PATH approaches, Dr. James Chow, an orthopaedic surgeon in Phoenix, Arizona, United States, combined the salient components of both approaches to develop a new approach in 2011, the SuperPATH (Supercapsular Percutaneously-Assisted Total Hip) [34, 35]. The SuperPATH combined the capsulotomy in the interval between piriformis and gluteus medius, and the technique of in situ broaching of the femur of the SuperCap with the cannula assisted acetabular reaming of the PATH.

The direct superior approach (DSA) is another superior approach, introduced in 2011 by Dr. Douglas Roger and David Hill, both working in Palm Springs, California, United States [36]. This approach is a single-incision modification to the PATH technique described above. In this modification, angled reamers were re-introduced allowing the use of a single straight incision. A specialized short-stemmed, high angled reamer and customized tissue friendly rounded retractors were developed for this approach. Both (DSA and PATH) posterosuperior approaches preserve the iliotibial band and external rotators of the hip except for the piriformis and conjoined tendon (Fig. 3c) [33, 36]. Of the superior approaches described here, the DSA technique is probably most easily converted intraoperatively to a more standard posterior hip approach, providing a better overview in more difficult cases (e.g. patients with higher BMI) or in revision surgery [36].

## Discussion

The evolution of the posterior approach in hip surgery started with the use of a singular straight incision of Dr. Bernhard von Langenbeck (1810–1887) to remove only the femoral head in injured soldiers [2]. He believed his approach was the least harmful for the patient and surgeon. More invasive approaches of Kocher [10], Gibson [15], Moore [17] and the Judet brothers [20] were introduced to improve visualization of the hip during femoral head resection, management of femoral, pelvic or acetabular fractures, and the implementation of total hip arthroplasty. While extensive posterior approaches remain immensely popular for trauma surgery, minimally invasive techniques made their appearance after 1990, especially with total hip arthroplasty in mind. Mainly through the development of modern retractors, and cannulated or angled reamers, the minimally invasive techniques culminated into the superior approaches by Murphy [32], Penenenberg [33], Chow [34, 35] and Roger [36]. Direct visualization of all necessary landmarks and structures is herewith still available in a less invasive approach. While some critics warn of potential complications in minimally invasive approaches [37], especially during the early learning phase, von Langenbeck puts matters in perspective, emphasizing that the choice of method is less important to him than the careful and skilled performance of the procedure [7].

Modern minimally invasive superior approaches share many similarities to von Langenbeck’s ‘*longitudinal incision*’. The patient is put in the lateral cubitus position, with the hip flexed 45 degrees. In all these approaches, the basic incision is a straight one directed from the superior border of the trochanter toward the posterior iliac spine sparing the iliotibial band. Von Langenbeck was already aware of the importance of preserving the iliotibial band. His students frequently praised the extreme precision and skill with which he handled the splitting of the gluteal muscles, among other things [3]. Von Langenbeck still separated the gluteal muscles along with the other muscles attached to the trochanter. The relatively young war patients allowed him to do this while sparing the periosteal origins, potentially allowing some regeneration. Conversely, current superior approaches preserve as much as possible the anatomy and function of the gluteal muscles and external rotators. In von Langenbeck’s approach, as well as in the superior approaches, a straight capsular incision is made just above or beneath the piriformis tendon, depending on the need for in situ broaching. If visibility is impaired, the von Langenbeck or superior approach can simply be extended distally into the iliotibial band to allow a more extensive posterior exposure. Similarly, the base incision can be used in future more extensive procedures such as revision surgeries. The superior approaches essentially aim to be as least invasive as possible, much the same as von Langenbeck attempted more than a century and a half ago.

Evolution—whether biological or surgical—is a dynamic, iterative process in which modification build upon the foundations laid by successful predecessors. Just as species adapt over time, surgical approaches evolve through incremental refinements, often driven by the limitations of earlier techniques. Technological advancement in medicine rarely occurs as a sudden revolution; rather, it is a series of purposeful, cumulative improvements made as we “stand on the shoulders of giants.”

Occasionally, progress involves revisiting the past, recognizing that some early innovations possess enduring value. Von Langenbeck, motivated by dissatisfaction with existing methods, pioneered a novel approach. Kocher expanded on this foundation by extending into the iliotibial band. Moore and the Judet brothers then refined and popularized the Kocher–Langenbeck approach, shaping it into the versatile, widely adopted technique known today.

However, evolution is not always linear. The advent of modern superior approaches reflects a return to core principles first introduced by von Langenbeck, now enhanced by contemporary imaging and instrumentation. In this sense, the posterior approach has come full circle. Ultimately, surgical progress is not about discarding the past but rediscovering its wisdom – “*Back to Langenbeck*”.

## Data Availability

No datasets were generated or analysed during the current study.
